# Crystal structure of tetra­kis­(μ-*n*-butyrato-κ^2^ *O*:*O*′)bis­[chlorido­rhenium(III)](*Re*—*Re*)

**DOI:** 10.1107/S1600536814020273

**Published:** 2014-09-13

**Authors:** Michael P. Thomson, Natasha F. O’Rourke, Ruiyao Wang, Manuel A. S. Aquino

**Affiliations:** a Department of Chemistry, St. Francis Xavier University, PO Box 5000, Antigonish, Nova Scotia, B2G 2W5, Canada; b Department of Chemistry, Queen’s University, Kingston, Ontario, Canada, K7L 3N6

**Keywords:** crystal structure, dirhenium core, butyrate bridging ligand

## Abstract

With an inversion center at the mid-point of the two Re^III^ atoms, the title compound, [Re_2_Cl_2_{O_2_C(CH_2_)_2_CH_3_}_4_], exhibits a paddle-wheel or lantern-type structure with four *n*-butyrate groups bridging two Re^III^ atoms in a *syn–syn* fashion. The axial chloride ligands together with the Re—Re quadruple bond [2.2330 (3) Å] complete an essentially octa­hedral geometry around each Re^III^ atom. There is little distortion, with an Re—Re—Cl bond angle of 176.18 (3)° and typical *cis*-O—Re—O bond angles ranging from 89.39 (11) to 90.68 (11)°. There are two mol­ecules in the unit cell, and no significant inter­molecular inter­actions were noticed between mol­ecules in the crystal.

## Related literature   

For the synthesis and structure of five related structures, see: Taha & Wilkinson (1963 ▶); Calvo *et al.* (1969 ▶); Collins *et al.* (1979 ▶); Lydon *et al.* (2003 ▶).
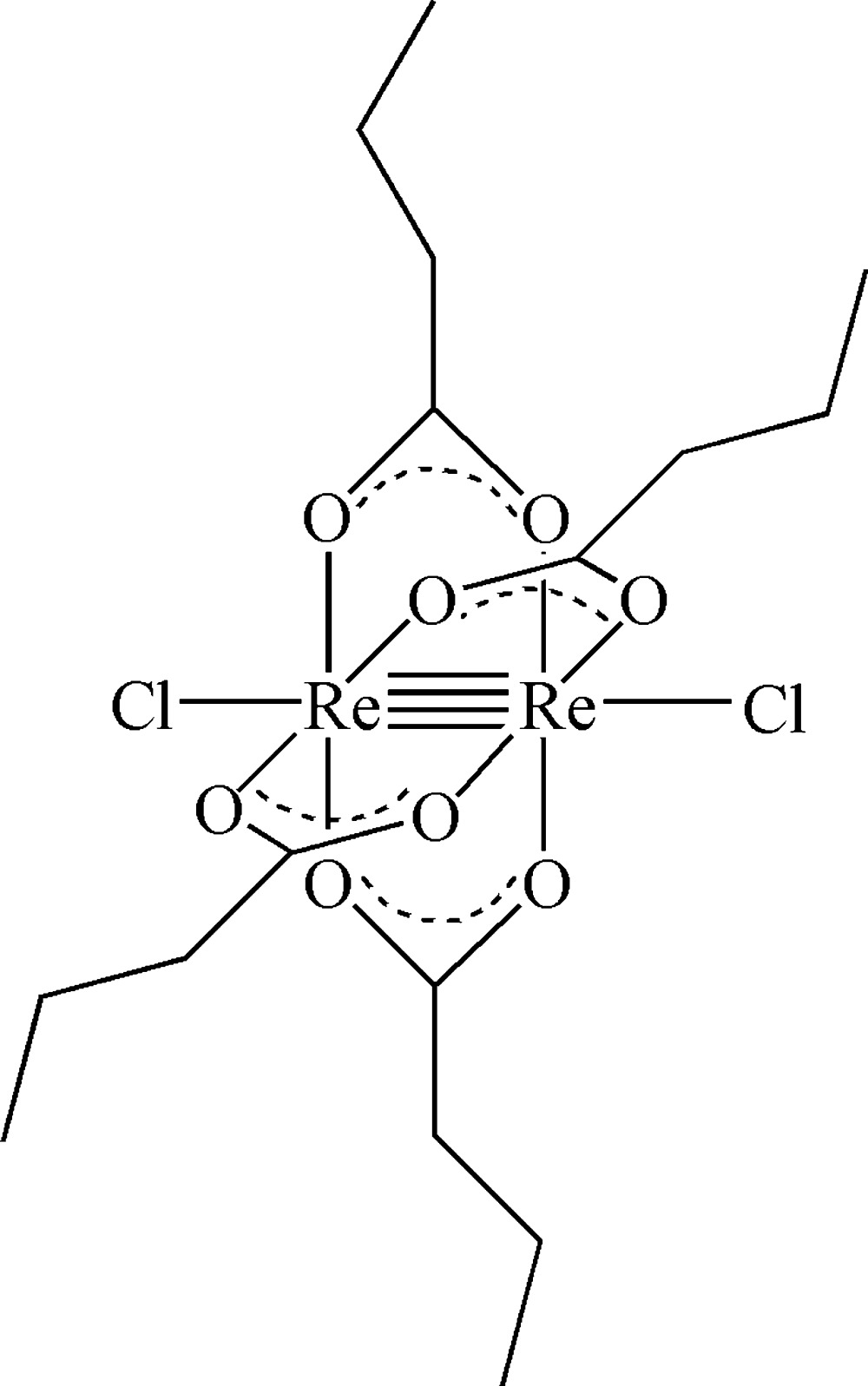


## Experimental   

### Crystal data  

[Re_2_(C_4_H_7_O_2_)_4_Cl_2_]
*M* *_r_* = 791.68
Monoclinic, 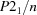

*a* = 6.7292 (4) Å
*b* = 12.0367 (8) Å
*c* = 14.6737 (9) Å
β = 99.262 (1)°
*V* = 1173.0 (1) Å^3^
*Z* = 2
Mo *K*α radiation
μ = 10.57 mm^−1^
*T* = 180 K
0.30 × 0.06 × 0.06 mm

### Data collection  

Bruker APEXII CCD diffractometer
Absorption correction: multi-scan (*SADABS*; Bruker, 2010 ▶) *T*_min_ = 0.144, *T*_max_ = 0.570
4625 measured reflections
2277 independent reflections
2077 reflections with *I* > 2σ(*I*)
*R*_int_ = 0.016

### Refinement  

*R*[*F* ^2^ > 2σ(*F* ^2^)] = 0.019
*wR*(*F* ^2^) = 0.049
*S* = 1.06
2277 reflections
129 parameters
H-atom parameters constrained
Δρ_max_ = 0.90 e Å^−3^
Δρ_min_ = −1.32 e Å^−3^

### 

Data collection: *APEX2* (Bruker, 2010 ▶); cell refinement: *SAINT* (Bruker, 2010 ▶); data reduction: *SAINT*; program(s) used to solve structure: *SHELXS97* (Sheldrick, 2008 ▶); program(s) used to refine structure: *SHELXL97* (Sheldrick, 2008 ▶); molecular graphics: *SHELXTL* (Sheldrick, 2008 ▶); software used to prepare material for publication: *SHELXTL*.

## Supplementary Material

Crystal structure: contains datablock(s) I, New_Global_Publ_Block. DOI: 10.1107/S1600536814020273/wm5055sup1.cif

**Fig. 1.**
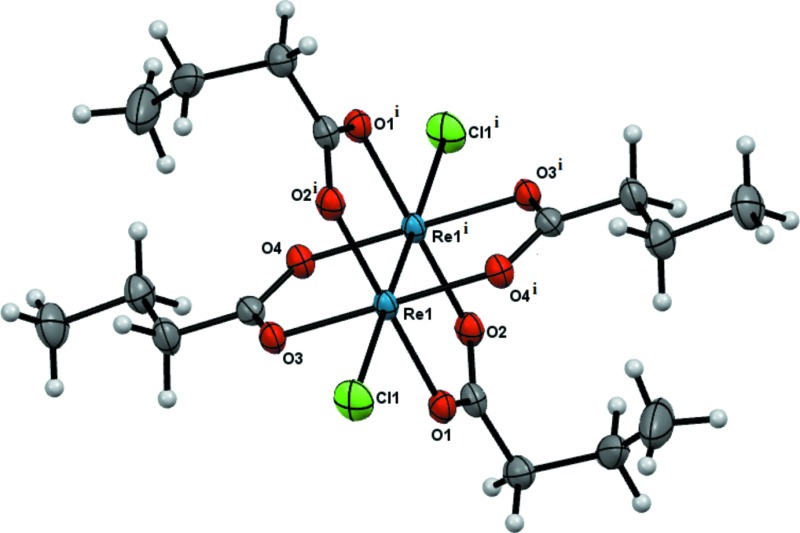
The molecular structure of the title compound with displacement ellipsoids drawn at the 50% probability level. Hydrogen atoms are drawn as small spheres of arbitrary radius. [Symmetry code i) -x + 2, -y + 1, -z + 2.]

Structure factors: contains datablock(s) I. DOI: 10.1107/S1600536814020273/wm5055Isup2.hkl

x y z . DOI: 10.1107/S1600536814020273/wm5055fig1.tif
The mol­ecular structure of the title compound with displacement ellipsoids drawn at the 50% probability level. Hydrogen atoms are drawn as small spheres of arbitrary radius. [Symmetry code i) −*x* + 2, −*y* + 1, −*z* + 2.]

**Fig. 2.**
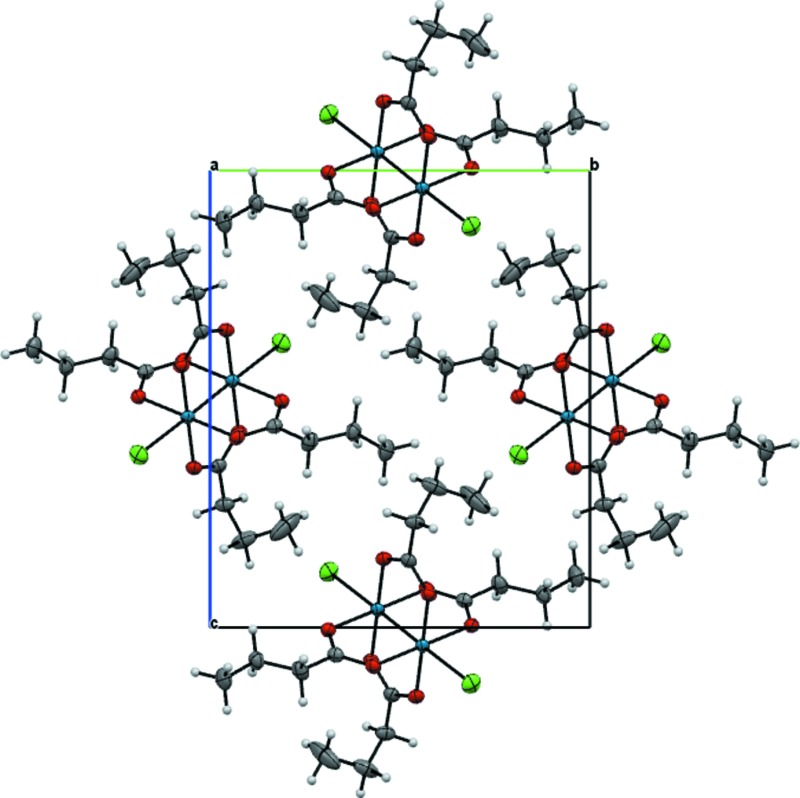
The packing diagram for the title compound viewed along [100].

. DOI: 10.1107/S1600536814020273/wm5055fig2.tif
The packing diagram for the title compound viewed along [100].

CCDC reference: 1023523

Additional supporting information:  crystallographic information; 3D view; checkCIF report

## Figures and Tables

**Table 1:** Selected bond lengths (Å)

Re1—O2^i^	2.008 (3)
Re1—O3	2.019 (2)
Re1—O4^i^	2.025 (2)
Re1—O1	2.026 (3)
Re1—Cl1	2.5135 (9)

Symmetry code: (i) 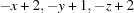.

## supplementary crystallographic information

### S1. Synthesis and crystallization

This compund was prepared following a method analogous to the procedure used by Taha & Wilkinson (1963). Rhenium trichloride (1.25 g, 4.27 mmol) was reacted with 13 ml of butyric acid and 0.5 ml of butyric anhydride at 423 K, with stirring, under a steady stream of nitro­gen for five days. The dark solution was removed from heat and allowed to cool overnight. The brown product was collected *via* suction filtration and washed with a 50 ml portion of petroleum ether. The product was dried *in vacuo* for 24 hours and then re-crystallized from di­chloro­methane. Yield = 0.481g, (56.9 %).

### S2. Refinement

All H atoms were placed in geometrically calculated positions, with C—H = 0.99 Å (CH_2_), and 0.98 Å (CH_3_), and refined as riding atoms, with *U*_iso_(H) = 1.5*U*_eq_(C) (CH_3_) or 1.2*U*_eq_(C) (CH_2_).

### Crystal data

**Table:**

[Re_2_(C_4_H_7_O_2_)_4_Cl_2_]	*F*(000) = 744
*M**_r_* = 791.68	*D*_x_ = 2.241 Mg m^−^^3^
Monoclinic, *P*2_1_/*n*	Mo *K*α radiation, λ = 0.71073 Å
Hall symbol: -P 2yn	Cell parameters from 3188 reflections
*a* = 6.7292 (4) Å	θ = 3.7–27.1°
*b* = 12.0367 (8) Å	µ = 10.57 mm^−^^1^
*c* = 14.6737 (9) Å	*T* = 180 K
β = 99.262 (1)°	Needle, orange
*V* = 1173.0 (1) Å^3^	0.30 × 0.06 × 0.06 mm
*Z* = 2	

### Data collection

**Table:**

Bruker APEXII CCD diffractometer	2277 independent reflections
Radiation source: fine-focus sealed tube	2077 reflections with *I* > 2σ(*I*)
Graphite monochromator	*R*_int_ = 0.016
φ and ω scans	θ_max_ = 26.0°, θ_min_ = 3.7°
Absorption correction: multi-scan (*SADABS*; Bruker, 2010)	*h* = −8→7
*T*_min_ = 0.144, *T*_max_ = 0.570	*k* = −12→14
4625 measured reflections	*l* = −18→18

### Refinement

**Table:**

Refinement on *F*^2^	Primary atom site location: structure-invariant direct methods
Least-squares matrix: full	Secondary atom site location: difference Fourier map
*R*[*F*^2^ > 2σ(*F*^2^)] = 0.019	Hydrogen site location: inferred from neighbouring sites
*wR*(*F*^2^) = 0.049	H-atom parameters constrained
*S* = 1.06	*w* = 1/[σ^2^(*F*_o_^2^) + (0.0278*P*)^2^ + 0.2053*P*] where *P* = (*F*_o_^2^ + 2*F*_c_^2^)/3
2277 reflections	(Δ/σ)_max_ = 0.005
129 parameters	Δρ_max_ = 0.90 e Å^−^^3^
0 restraints	Δρ_min_ = −1.32 e Å^−^^3^

### Special details

**Table:**

Geometry. All e.s.d.'s (except the e.s.d. in the dihedral angle between two l.s. planes) are estimated using the full covariance matrix. The cell e.s.d.'s are taken into account individually in the estimation of e.s.d.'s in distances, angles and torsion angles; correlations between e.s.d.'s in cell parameters are only used when they are defined by crystal symmetry. An approximate (isotropic) treatment of cell e.s.d.'s is used for estimating e.s.d.'s involving l.s. planes.
Refinement. Refinement of *F*^2^ against ALL reflections. The weighted *R*-factor *wR* and goodness of fit *S* are based on *F*^2^, conventional *R*-factors *R* are based on *F*, with *F* set to zero for negative *F*^2^. The threshold expression of *F*^2^ > σ(*F*^2^) is used only for calculating *R*-factors(gt) *etc*. and is not relevant to the choice of reflections for refinement. *R*-factors based on *F*^2^ are statistically about twice as large as those based on *F*, and *R*- factors based on ALL data will be even larger.

### Fractional atomic coordinates and isotropic or equivalent isotropic displacement parameters (Å^2^)

**Table:**

	*x*	*y*	*z*	*U*_iso_*/*U*_eq_	
Re1	0.91824 (2)	0.559012 (11)	1.039538 (10)	0.02050 (7)	
Cl1	0.71261 (17)	0.68750 (8)	1.12219 (8)	0.0397 (3)	
O1	0.7020 (4)	0.57529 (19)	0.92707 (18)	0.0254 (6)	
O2	0.8665 (4)	0.45750 (19)	0.84940 (19)	0.0256 (6)	
O3	0.7697 (4)	0.42938 (19)	1.08438 (19)	0.0243 (6)	
O4	0.9312 (4)	0.31123 (19)	1.00486 (18)	0.0241 (6)	
C1	0.7167 (6)	0.5223 (3)	0.8532 (3)	0.0253 (8)	
C2	0.5652 (6)	0.5386 (3)	0.7691 (3)	0.0308 (9)	
H2A	0.4565	0.5877	0.7838	0.037*	
H2B	0.5045	0.4661	0.7486	0.037*	
C3	0.6593 (7)	0.5899 (4)	0.6916 (3)	0.0451 (12)	
H3A	0.7561	0.5366	0.6719	0.054*	
H3B	0.5526	0.6040	0.6380	0.054*	
C4	0.7670 (9)	0.6974 (5)	0.7202 (5)	0.083 (2)	
H4A	0.8092	0.7325	0.6662	0.125*	
H4B	0.8857	0.6821	0.7666	0.125*	
H4C	0.6761	0.7474	0.7464	0.125*	
C5	0.8023 (6)	0.3306 (3)	1.0595 (3)	0.0247 (8)	
C6	0.6899 (6)	0.2376 (3)	1.0943 (3)	0.0338 (10)	
H6A	0.5453	0.2464	1.0688	0.041*	
H6B	0.7037	0.2445	1.1623	0.041*	
C7	0.7539 (7)	0.1221 (3)	1.0718 (3)	0.0333 (9)	
H7A	0.8978	0.1111	1.0975	0.040*	
H7B	0.7373	0.1126	1.0040	0.040*	
C8	0.6260 (8)	0.0359 (3)	1.1127 (3)	0.0426 (11)	
H8A	0.6674	−0.0389	1.0974	0.064*	
H8B	0.4836	0.0470	1.0871	0.064*	
H8C	0.6449	0.0445	1.1800	0.064*	

### Atomic displacement parameters (Å^2^)

**Table:**

	*U*^11^	*U*^22^	*U*^33^	*U*^12^	*U*^13^	*U*^23^
Re1	0.02322 (11)	0.02022 (10)	0.02042 (10)	0.00156 (6)	0.01068 (7)	0.00100 (5)
Cl1	0.0449 (6)	0.0377 (5)	0.0419 (6)	0.0105 (5)	0.0232 (5)	−0.0039 (5)
O1	0.0283 (15)	0.0278 (13)	0.0217 (14)	0.0015 (11)	0.0092 (12)	0.0030 (10)
O2	0.0294 (16)	0.0275 (13)	0.0222 (14)	0.0013 (11)	0.0108 (12)	−0.0003 (10)
O3	0.0256 (15)	0.0252 (13)	0.0243 (14)	−0.0007 (10)	0.0105 (12)	0.0029 (10)
O4	0.0267 (14)	0.0235 (13)	0.0238 (13)	−0.0025 (11)	0.0086 (11)	−0.0006 (11)
C1	0.027 (2)	0.0276 (18)	0.024 (2)	−0.0031 (16)	0.0112 (17)	0.0024 (15)
C2	0.029 (2)	0.041 (2)	0.024 (2)	0.0023 (18)	0.0081 (17)	0.0013 (17)
C3	0.034 (3)	0.071 (3)	0.033 (2)	0.015 (2)	0.012 (2)	0.021 (2)
C4	0.063 (4)	0.095 (5)	0.084 (5)	−0.027 (4)	−0.010 (3)	0.060 (4)
C5	0.0217 (19)	0.0284 (19)	0.0246 (19)	−0.0020 (16)	0.0058 (15)	0.0014 (15)
C6	0.035 (2)	0.031 (2)	0.039 (2)	−0.0015 (18)	0.0177 (19)	0.0082 (17)
C7	0.038 (2)	0.025 (2)	0.038 (2)	−0.0064 (17)	0.011 (2)	0.0019 (17)
C8	0.048 (3)	0.031 (2)	0.049 (3)	−0.012 (2)	0.012 (2)	0.0063 (19)

### Geometric parameters (Å, º)

**Table:**

Re1—O2^i^	2.008 (3)	C3—C4	1.509 (7)
Re1—O3	2.019 (2)	C3—H3A	0.9900
Re1—O4^i^	2.025 (2)	C3—H3B	0.9900
Re1—O1	2.026 (3)	C4—H4A	0.9800
Re1—Re1^i^	2.2330 (3)	C4—H4B	0.9800
Re1—Cl1	2.5135 (9)	C4—H4C	0.9800
O1—C1	1.275 (5)	C5—C6	1.487 (5)
O2—C1	1.283 (4)	C6—C7	1.508 (5)
O2—Re1^i^	2.008 (3)	C6—H6A	0.9900
O3—C5	1.273 (4)	C6—H6B	0.9900
O4—C5	1.294 (4)	C7—C8	1.531 (5)
O4—Re1^i^	2.025 (2)	C7—H7A	0.9900
C1—C2	1.482 (6)	C7—H7B	0.9900
C2—C3	1.520 (6)	C8—H8A	0.9800
C2—H2A	0.9900	C8—H8B	0.9800
C2—H2B	0.9900	C8—H8C	0.9800
			
O2^i^—Re1—O3	89.39 (11)	C4—C3—H3B	109.2
O2^i^—Re1—O4^i^	90.26 (10)	C2—C3—H3B	109.2
O3—Re1—O4^i^	179.64 (11)	H3A—C3—H3B	107.9
O2^i^—Re1—O1	179.70 (10)	C3—C4—H4A	109.5
O3—Re1—O1	90.68 (11)	C3—C4—H4B	109.5
O4^i^—Re1—O1	89.67 (10)	H4A—C4—H4B	109.5
O2^i^—Re1—Re1^i^	90.42 (7)	C3—C4—H4C	109.5
O3—Re1—Re1^i^	89.36 (7)	H4A—C4—H4C	109.5
O4^i^—Re1—Re1^i^	90.55 (7)	H4B—C4—H4C	109.5
O1—Re1—Re1^i^	89.29 (7)	O3—C5—O4	120.7 (3)
O2^i^—Re1—Cl1	92.90 (8)	O3—C5—C6	118.9 (3)
O3—Re1—Cl1	88.76 (8)	O4—C5—C6	120.4 (3)
O4^i^—Re1—Cl1	91.34 (7)	C5—C6—C7	116.1 (3)
O1—Re1—Cl1	87.40 (8)	C5—C6—H6A	108.3
Re1^i^—Re1—Cl1	176.18 (3)	C7—C6—H6A	108.3
C1—O1—Re1	120.0 (2)	C5—C6—H6B	108.3
C1—O2—Re1^i^	119.6 (2)	C7—C6—H6B	108.3
C5—O3—Re1	120.7 (2)	H6A—C6—H6B	107.4
C5—O4—Re1^i^	118.7 (2)	C6—C7—C8	109.9 (4)
O1—C1—O2	120.7 (4)	C6—C7—H7A	109.7
O1—C1—C2	120.3 (3)	C8—C7—H7A	109.7
O2—C1—C2	119.0 (3)	C6—C7—H7B	109.7
C1—C2—C3	111.3 (4)	C8—C7—H7B	109.7
C1—C2—H2A	109.4	H7A—C7—H7B	108.2
C3—C2—H2A	109.4	C7—C8—H8A	109.5
C1—C2—H2B	109.4	C7—C8—H8B	109.5
C3—C2—H2B	109.4	H8A—C8—H8B	109.5
H2A—C2—H2B	108.0	C7—C8—H8C	109.5
C4—C3—C2	112.3 (4)	H8A—C8—H8C	109.5
C4—C3—H3A	109.2	H8B—C8—H8C	109.5
C2—C3—H3A	109.2		
			
O2^i^—Re1—O1—C1	13 (19)	Re1^i^—O2—C1—O1	−0.9 (5)
O3—Re1—O1—C1	−89.7 (3)	Re1^i^—O2—C1—C2	176.6 (2)
O4^i^—Re1—O1—C1	90.2 (3)	O1—C1—C2—C3	115.7 (4)
Re1^i^—Re1—O1—C1	−0.3 (3)	O2—C1—C2—C3	−61.7 (5)
Cl1—Re1—O1—C1	−178.4 (3)	C1—C2—C3—C4	−55.2 (5)
O2^i^—Re1—O3—C5	−90.7 (3)	Re1—O3—C5—O4	−0.1 (5)
O4^i^—Re1—O3—C5	−77 (17)	Re1—O3—C5—C6	179.9 (3)
O1—Re1—O3—C5	89.0 (3)	Re1^i^—O4—C5—O3	0.5 (5)
Re1^i^—Re1—O3—C5	−0.3 (3)	Re1^i^—O4—C5—C6	−179.5 (3)
Cl1—Re1—O3—C5	176.4 (3)	O3—C5—C6—C7	−172.7 (4)
Re1—O1—C1—O2	0.8 (5)	O4—C5—C6—C7	7.2 (6)
Re1—O1—C1—C2	−176.6 (2)	C5—C6—C7—C8	179.5 (4)

Symmetry code: (i) −*x*+2, −*y*+1, −*z*+2.
