# Supplementary figures and images for: Crystal structure of tetra­kis­(μ-n-butyrato-κ2 O:O′)bis­[chlorido­rhenium(III)](Re—Re)
Source: Acta Crystallogr Sect E Struct Rep Online. 2014 Sep 13;70(Pt 10):m349–50. doi: 10.1107/S1600536814020273 (PMC4257215; doi:10.1107/S1600536814020273)

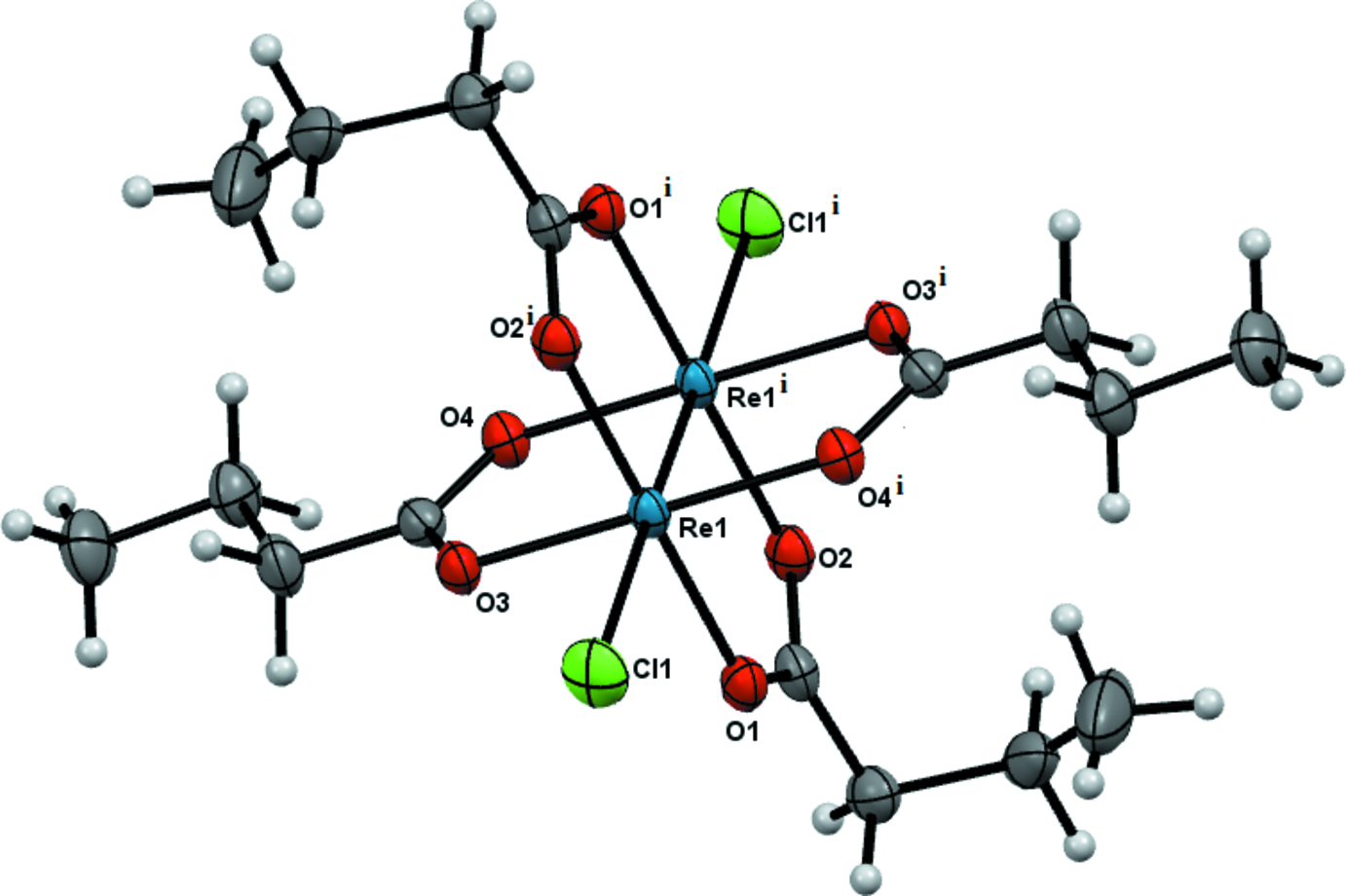

Supplement: Supplementary file 3 [file e-70-0m349-fig1.tif]

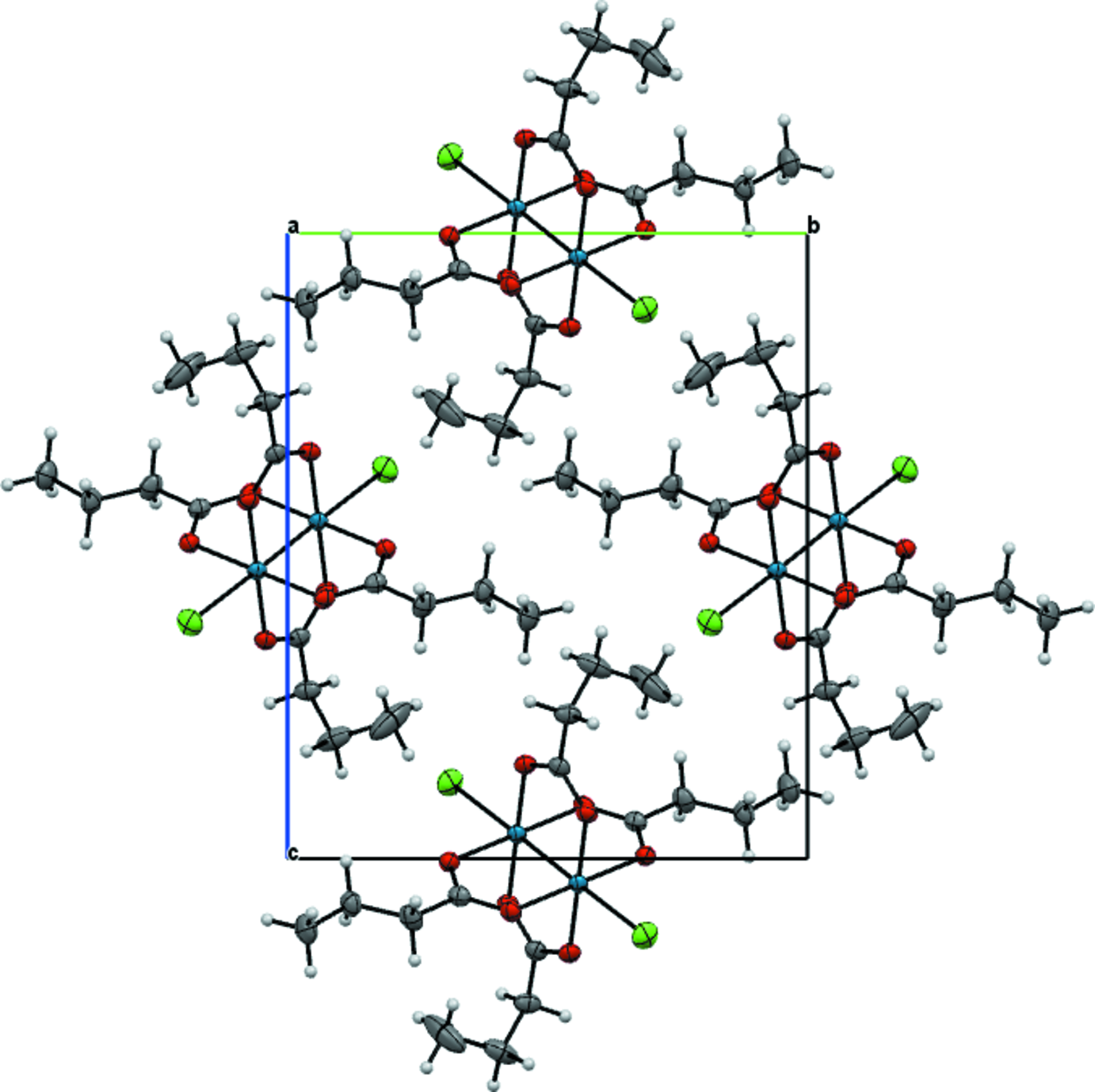

Supplement: Supplementary file 4 [file e-70-0m349-fig2.tif]
